# Common themes and cell type specific variations of higher order chromatin arrangements in the mouse

**DOI:** 10.1186/1471-2121-6-44

**Published:** 2005-12-07

**Authors:** Robert Mayer, Alessandro Brero, Johann von Hase, Timm Schroeder, Thomas Cremer, Steffen Dietzel

**Affiliations:** 1 Ludwig-Maximilians-Universität München, Department Biologie II, Groβhaderner Str 2, 82152 Planegg-Martinsried, Germany; 2 Kirchhoff Institut für Physik, Universität Heidelberg, Germany; 3 Institute of Stem Cell Research, GSF – National Research Center for Environment and Health, Neuherberg, Germany

## Abstract

**Background:**

Similarities as well as differences in higher order chromatin arrangements of human cell types were previously reported. For an evolutionary comparison, we now studied the arrangements of chromosome territories and centromere regions in six mouse cell types (lymphocytes, embryonic stem cells, macrophages, fibroblasts, myoblasts and myotubes) with fluorescence in situ hybridization and confocal laser scanning microscopy. Both species evolved pronounced differences in karyotypes after their last common ancestors lived about 87 million years ago and thus seem particularly suited to elucidate common and cell type specific themes of higher order chromatin arrangements in mammals.

**Results:**

All mouse cell types showed non-random correlations of radial chromosome territory positions with gene density as well as with chromosome size. The distribution of chromosome territories and pericentromeric heterochromatin changed during differentiation, leading to distinct cell type specific distribution patterns. We exclude a strict dependence of these differences on nuclear shape. Positional differences in mouse cell nuclei were less pronounced compared to human cell nuclei in agreement with smaller differences in chromosome size and gene density. Notably, the position of chromosome territories relative to each other was very variable.

**Conclusion:**

Chromosome territory arrangements according to chromosome size and gene density provide common, evolutionary conserved themes in both, human and mouse cell types. Our findings are incompatible with a previously reported model of parental genome separation.

## Background

The existence of chromosome territories as restricted volumes in which the DNA of only one chromosome is spatially arranged during interphase is now established for about 20 years [1,2]. The distribution of individual territories within the nucleus has come into focus more recently. Although the side-by-side arrangement of chromosome territories can change from one cell cycle to the next [3,4], the radial organization of chromatin in the nucleus in general is not random. So far, the most widespread detected principle of functional nuclear architecture is the specific positioning of chromatin with different replication time points in S-phase. From single cell eukaryotes [5] to distantly related multicellular organisms like Hydra [6], chicken [7], humans [8,9] and plants [10], a layer of chromatin replicating in mid S-phase was found at the nuclear periphery and around nucleoli, while early replicating chromatin was distributed in interior nuclear zones between the perinucleolar and perinuclear compartments.

For chromosome territories and some chromosomal subregions non-random radial distributions have been described. Several studies have shown that in spherical nuclei of both quiescent and cycling human lymphocytes, gene rich chromosomes are statistically more often centrally located while gene poor chromosomes are preferentially at the periphery [11-13]. Such an arrangement has also been reported for the gene rich human chromosome 19 homologs and gene poor chromosome 18 homologs in other primates [14]. Controversial results have been reported for flat human fibroblast nuclei. While one group described gene rich chromosome territories to be centrally and gene poor ones peripherally located [11,13,15], other groups described a size dependent radial distribution where large chromosomes are preferentially peripheral and small chromosomes internal [12,16,17]. While for spherical nuclei of cells growing in suspension all sites at the nuclear periphery are topologically indistinguishable from each other, a flat ellipsoidal nucleus as in fibroblasts possesses a unique outer rim defined by the intersection of the horizontal mid-plane with the nuclear border. In recent work on fibroblast nuclei of *Homo sapiens *(HSA) we found that territories of both, gene poor chromosome HSA18 and gene rich chromosome HSA19, stay close to the nuclear center, remote from the outer rim just described [17]. Accordingly, they were often neighbors. HSA18 territories, however, were on average located closer than HSA19 territories to the top and bottom part of the envelope of structurally preserved nuclei. In contrast, in spherical lymphocyte nuclei gene rich HSA19 territories are typically located in the nuclear interior while gene poor HSA18 territories are associated with the nuclear envelope and thus away from HSA19. The shape of nuclei thus apparently plays a role in territory positioning. Differences in the distribution of some chromosome territories in different cell types have also been described in mouse [18]. Interestingly, cell types from related differentiation pathways like large and small lung cells, were found to have more similar chromosomal distribution patterns than unrelated cell types. Only chromosomes with low to average gene density from unsynchronized cells were investigated [18] and gene density and cell cycle topics were not addressed. In chicken, large, gene poor chromosomes have been found peripheral and small, gene rich ones more centrally in flat fibroblast nuclei, in semi-spherical neuroblast nuclei and spherical nuclei of some blood cell types [7,19].

While repetitive probes for individual centromeric regions have been used in many studies to identify the position of specific chromosomes [20-22] centromeres as functional entities also have been a subject of interest. Changes in centromere distribution have been associated with differentiation processes [23-26]. Clusters of centromeric and pericentromeric regions, so called chromocenters, were shown to be involved in silencing of several genes in various hematopoietic cell types [27,28] and MeCP2, a protein binding to methylated DNA, was shown to induce clustering of these regions during mouse myotube differentiation [29]. These findings indicate that in order to understand the rules which govern nuclear functions, we need to understand chromatin structure at all levels, from the organization of the nucleosome fiber carrying individual active and silent genes to the architecture and arrangements of chromosome territories in the nuclear space.

**Figure 1:**
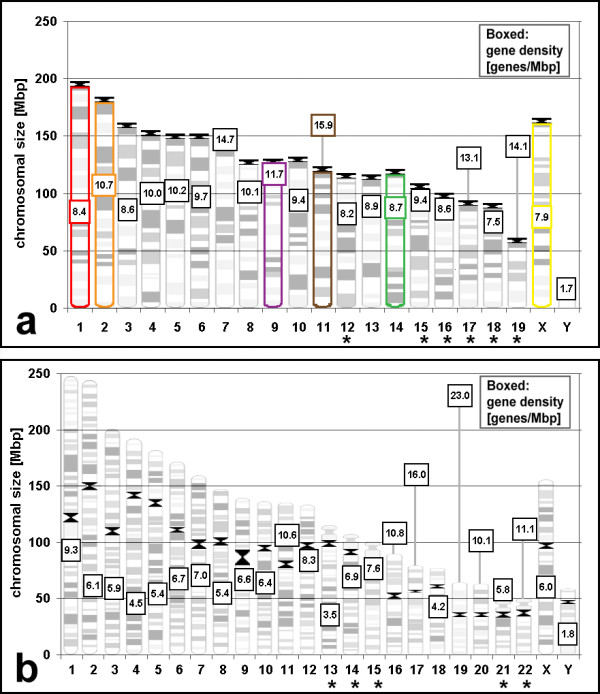
Sizes and gene densities of mouse (a) and human (b) chromosomes. The length of chromosomes is scaled according to their genomic size. Gene density in genes/Mbp for each chromosome is indicated in a box. The distance of the box to the x-axis is scaled according to the gene density. Centromeres are indicated in black. Chromosomes with NORs are indicated with an asterisk. Mouse chromosomes investigated in this study are surrounded with the color used in Figure 3 for their representation. (a) Mouse chromosomes have an average size of 124 ± 32 Mbp (standard deviation) and an average gene density of 10.1 ± 2.3 genes/Mbp. For both parameters the variation in human chromosomes (b) is thus much larger. They have an average size of 128 ± 56 Mbp and an average gene density of 7.3 ± 4.2 genes/Mbp. Values are disregarding the Y-chromosomes. Data are from Ensemble Genome Browser [30].

In the present study we tested the spatial organization of chromocenters and chromosome territories for specific distribution patterns in six mouse cell types. We also asked whether chromosome distribution in lymphocytes and fibroblasts is conserved from man to mouse. An obvious difference between human and mouse karyotypes is that all chromosomes of *Mus musculus *have the centromere near one telomere (telocentric) with large heterochromatic blocks nearby (Figure 1a) while human chromosomes are divided by the centromere in two arms of approximately similar size (metacentric) or a rather short and rather long arm (acrocentric) or ratios in between (submetacentric) (Figure 1b). Differences in gene content and size between chromosomes are also smaller in mouse than in humans (Figure 1). Disregarding human and mouse Y-chromosomes, human chromosome sizes vary about fivefold (245 – 47 Megabasepairs, Mbp [30] and human chromosome gene densities vary more than sixfold (23 – 3.5 genes/Mbp [30]). Mouse chromosomes (Figure 1a) vary in gene density only about twofold (15.9 – 7.5 genes/Mbp) and in size about threefold (195 – 61 Mbp). We tested the radial distribution of mouse chromosome territories for size-dependence and gene-density dependence in cell types with nuclear shapes ranging from spherical to flat ellipsoidal. We also investigated the position of homologous and pairs of heterologous chromosomes relative to each other. We selected 6 chromosomes to cover big, small, gene rich and gene poor examples (Figure 1a). For chromocenters, the degree of clustering and the nuclear distribution was measured in each cell type. All examinations were made on nuclei with a defined cell cycle stage.

## Results

### Experimental design

**Figure 2:**
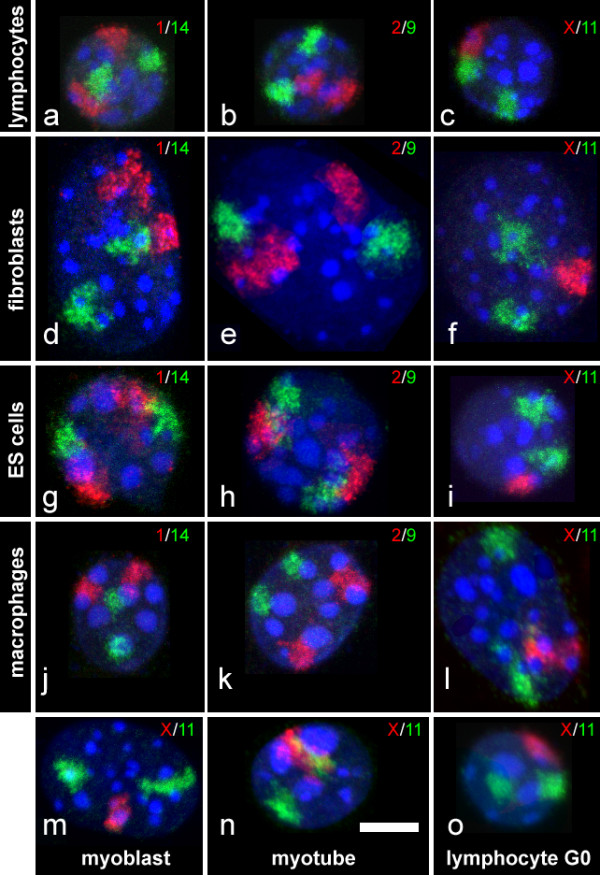
3D-FISH on structurally preserved mouse nuclei. Mouse chromosome pairs MMU1/MMU14 (a, d, g, j), MMU2/MMU9 (b, e, h, k) and MMU11/MMUX (c, f, i, l, m, n, o) were detected in S-phase lymphocytes (a, b, c), fibroblasts (d, e, f) embryonic stem cells (g,h,i), macrophages (j, k, l), myoblasts (m), myotubes (n) and G0 lymphocytes (o). TO-PRO-3 (pseudocolored blue) was used as DNA counterstain. Maximum intensity projections of confocal image stacks are shown. All images are shown to scale. Scalebar: 5 μm

We performed dual color fluorescence in situ hybridization on formaldehyde fixed, morphologically preserved nuclei (3D-FISH) of *Mus musculus *(MMU) cells (Figure 2). Embryonic stem (ES) cells, in vitro differentiated macrophages, primary fibroblasts and stimulated primary lymphocytes were hybridized with the following combinations of whole chromosome paint probes: chromosomes 1 and 14, 2 and 9, 11 and X. Chromosomes 11 and X were additionally hybridized to unstimultated lymphocytes, myoblasts and myotubes. The rationale for choosing these chromosomes reflects their differences in size and gene content (Figure 1). MMU1 is the largest mouse chromosome (195 Mbp). MMU14 is comparatively small (119 Mbp) and both are gene poor (8.4 and 8.7 genes/Mbp, respectively). MMU2 is large (182 Mbp) compared to MMU 9 (124 Mbp) and both are more gene rich than the first pair (10.7 and 11.7 genes/Mbp, respectively), being the 6^th ^and 5^th ^gene richest chromosome. MMU11 (122 Mbp) is similar in size to MMU9 and MMU14 and the most gene rich chromosome in the mouse karyotype (15.9 genes/Mbp). MMUX has the third lowest gene density of all mouse chromosomes (7.9 genes/Mbp) and is rather large (164 Mbp). Only X-chromosomes from male cells were investigated. The smaller chromosomes 15–19 were not included in our analysis since they as well as MMU12 may contain nucleolar organizing regions [31,32] which are tethered to nucleoli and thus spatial distribution would be biased.

To minimize potential influences of cell cycle stages [15,33-35] we only recorded nuclei in a defined stage. In proliferating cell types, cultures were labeled with a short BrdU pulse to identify S-phase cells, and only BrdU positive cells were recorded. Post-mitotic macrophages were identified by the absence of a BrdU signal after 24 hours of incubation. Unstimulated lymphocytes and myotubes were considered as G0 since in control experiments they did not incorporate BrdU even if incubation times >24 h were used. After counterstaining for DNA, confocal image stacks were recorded and quantitatively evaluated for the parameters described in the following sections. For each chromosome combination and cell type we typically recorded 30 nuclei. All quantitative evaluations were carried out in 3D.

### The radial distribution of chromosome territories is cell type specific

**Figure 3:**
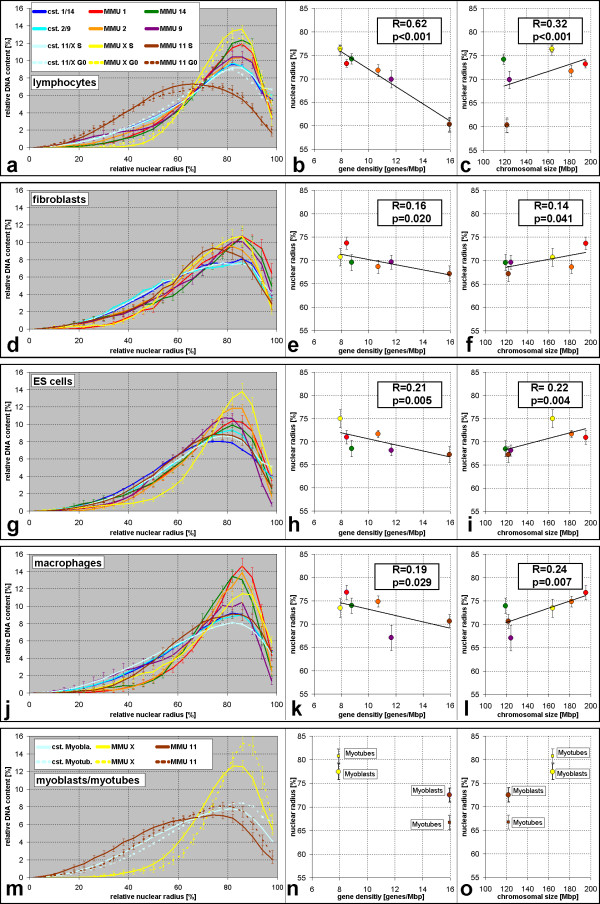
Three-dimensional relative radial nuclear distribution of mouse chromosome territories from a total of 481 nuclei with two labeled pairs of chromosomes each. Each nucleus was divided in 25 shells with equal spacing. The relative amount of the signal from a given chromosome paint probe or of the DNA counterstain (cst) in each of the shells was measured and averaged over all nuclei of the respective cell type (see Methods for details). Graphs on the left (a,d,g,j,m) show the percentage of each signal in the 25 shells. Error bars in (a-o) show the standard error of the mean. For each nucleus and territory signal, the median radius of the relative radial distribution was calculated (see additional data file 1 for complete listing). Graphs in the center (b,e,h,k,n) show the averages of these medians plotted against the gene density of the painted chromosome. In the graphs on the right (c,f,i,l,o) these averages of medians are plotted against chromosomal size. For cell types with investigated territories from six types of chromosomes, the black lines show the linear regression. The box shows the correlation coefficient R and the p-value indicating the probability that there is no correlation but that the observed relation was a chance result. Both values were calculated from individual medians from all nuclei while the regression line shown was fitted to the six average values of the medians represented by the dots. (a-c) Lymphocyte nuclei in S-phase (continuous lines in (a), circles in (b), all six investigated chromosomes) and in G0 (broken lines in (a), squares in b,c, MMU11 and MMUX only). In (b,c), data points for average medians in G0 lymphocytes fall exactly on data points in S-phase and are thus difficult to distinguish. G0 lymphocytes were not included in the linear regression analysis. (d-f) Fibroblast S-phase nuclei. (g-i) ES-cell S-phase nuclei. (j-l) Macrophage G0 nuclei. Color code shown in (a) also applies to (b-l). (m-o) Myoblast (continuous lines) and myotube nuclei (broken lines). Correlation coefficients for myoblasts (0.26, p = 0.032) and myotubes (0.68, p < 0.001) are not directly comparable since only two chromosomes were investigated. Note that in all cell types there is a correlation for gene poor as well as large chromosome territories to be more peripheral, although for some chromosome territories average medians diverge from this pattern.

**Figure 4:**
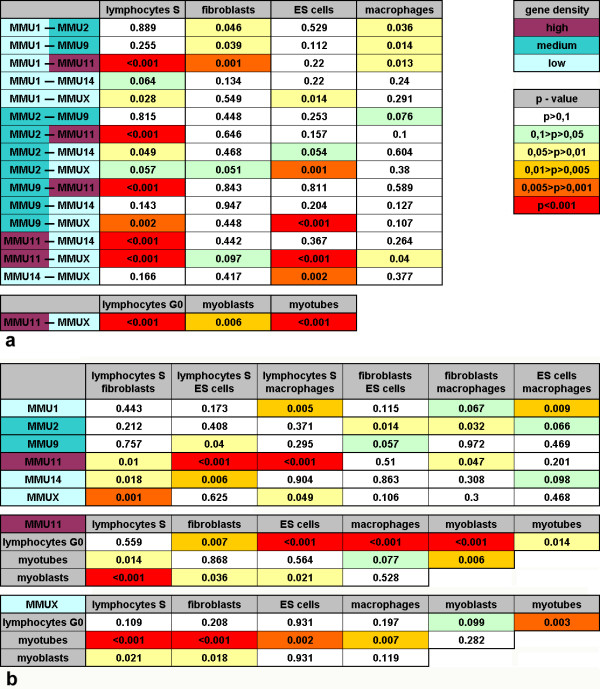
Statistical comparison of relative radial distributions of chromosome territories. Significance levels are shown as p-values from a two-sided Kolmogorov-Smirnov test and are color coded as indicated. Chromosome names are highlighted according to their gene density as indicated. (a) Pair wise comparison of the relative radial distributions of chromosomes in a given cell type. Lymphocytes in S-phase and in G0 are listed separately. (b) Pair wise comparison of the relative radial distributions of a given chromosome territory between cell types.

A total of 962 three-dimensional image stacks with chromosome territories plus the respective image stacks of counterstained nuclear DNA was analyzed. The radial distribution of painted chromosome territories was measured in each nucleus relative to the nuclear radius and averaged over the set of recorded nuclei (Figure 3). In mouse lymphocyte nuclei we found territories of the most gene rich chromosome MMU11 typically much more internally located than all other chromosomes, the most prominent difference in radial distribution in our study (see Figure 4 for p-values). MMU2 and MMU9 occupied intermediate positions and gene poor MMU1, MMU14 and MMUX were found more towards the periphery (Figure 3a,b,c,f). Thus the distribution of tested chromosomes was in agreement with a gene density related positioning. Linear regression analysis (Figure 3b) revealed a steeper line for lymphocyte nuclei than for other cell types, indicating a stronger dependency on this factor in this cell type. Linear regression resulted in a correlation coefficient of 0.62, indicating a good fit of the data (Figure 3b). The distribution of MMU11 in lymphocytes was significantly more internal compared to other cell types (see Figure 4 for p-values). No significant difference was found between G0 and S-phase lymphocytes.

Primary mouse fibroblasts differed from other cell types in that the radial distribution curves for all investigated chromosomes were rather similar (Figure 3d). A tendency for the location of gene rich chromosomes towards internal and of gene poor chromosomes towards the nuclear border was found (Figure 3e) but except for MMU1 differences between chromosomes were not significant (Figure 4). A tendency for a more peripheral location of large chromosomes was also noted (Figure 3f).

Although ES cell nuclei have a shape very similar to lymphocyte nuclei, some chromosome territories were distributed quite differently. MMU11 was located less internal than in lymphocyte nuclei (p < 0.001) although it was again the most internal chromosome (Figure 3g,h,i, Figure 4). The small, gene poor MMU14 territories were found significantly more internal than in lymphocyte nuclei. The correlation coefficient was similar for gene density and size dependent chromosome territory distribution. Post-mitotic macrophages were differentiated in vitro from ES-cells. They differed significantly from ES cells in the radial nuclear distribution of MMU1 which was now observed further outside (Figure 3j,k,l, Figure 4b). In this cell type, distribution of territories showed a better correlation with chromosomal size than with gene density (Figure 3k,l). MMU11 distribution changed significantly during myoblast-to-myotube differentiation (Figure 4b). MMUX territories were significantly more peripheral in S-phase myoblasts and in particular in postmitotic myotubes than in other cell types (Figure 3m,n,o, Figure 4b).

In a subset of nuclei from each cell type we visually analyzed whether chromosome territories were in contact with the nuclear border or not. In ES-cells (n = 28), macrophages (n = 28), myoblasts (n = 35) and myotubes (n = 21) all inspected MMU11 and MMUX territories had contact with the nuclear periphery. In 7 of 31 lymphocyte nuclei (23%), one of the two MMU11 homologs apparently did not touch the nuclear border while the other MMU11 and all MMUX territories did.

### Chromosome territories do not have fixed positions relative to each other

Confocal image stacks were also used to determine the positioning of chromosome territories relative to each other. Three-dimensional angles between chromosome territories were calculated using the intensity gravity centers of the chromosome territories and the geometrical center of the segmented nucleus as point of origin. If homologous chromosome territories were touching each other we attempted their separation by increasing the threshold. For those nuclei where a separation could not be achieved in this way, the angle between the homologs was set to zero.

**Figure 5:**
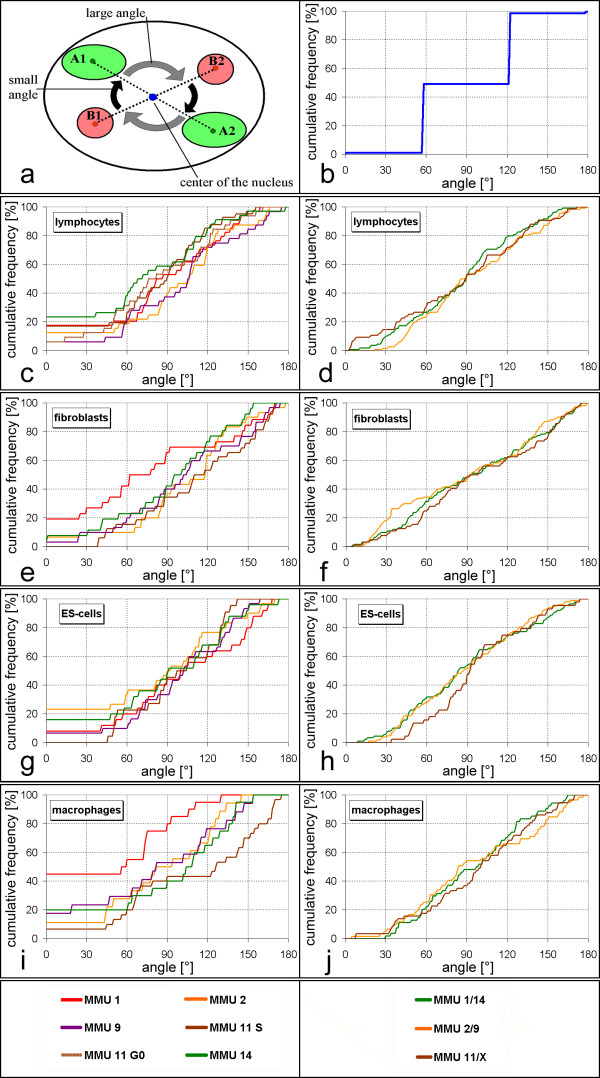
Angles between chromosome territories. (a) Scheme of fixed chromosomal angles between two pairs of chromosomes as predicted by the model of genome separation with antiparallel orientation [36]. In this example, specific angles are assumed to be about 60° and 120°. A cumulative distribution plot of homologous angles in this model has a steep increase from 0 to 100% near 180° (not shown). (b) Cumulative distribution of heterologous angles in this model example. For other chromosome pairs, angles and respective sharp increases of cumulative graphs would be at other values. Cumulative plots are shown for the indicated cell types. (c-j) Experimentally observed cumulative distributions of angles between homologous (c,e,g,i) or heterologous (d,f,h,j) chromosome territories in the indicated cell types.

Homologous association of chromosome territories would lead to small angles, while a parental separation of haploid sets with an antiparallel order of chromosomes as suggested by Nagele et al. [36] would lead to angles close to 180 degrees for homologs. In disagreement with both models, cumulative distribution curves show that angles cover the whole range between 0° and 180° for all investigated chromosomes in all investigated cell types (Figure 5c,e,g,i). While we observed a high frequency of 0° (= inseparable territories) in some cases, this was typically compensated by the sparse occurrence of small angles between 1° and 40°. An exception are MMU1 chromosomes in macrophages where 45% of the nuclei showed inseparable territories (9 of 20 nuclei). In the investigated cell types, only the comparison between MMU1 and MMU11 homologous association in macrophages revealed a highly significant difference (Table 1). Mean values also are not compatible with predictions of either model (Table 1). We conclude that measured angles between homologous chromosomes are incompatible with both, non-random homologous association and parental genome separation with the possible exception of MMU1 in macrophage nuclei.

**Table 1:** Angles between homologous chromosomes. Mean values (mv), median, standard deviation (std) and numbers of evaluated nuclei (n) are listed. In addition, p-values derived from the comparison of homologous angles between pairs of homologous chromosomes are shown. p-values <0.05 are underlined.

**lymphocytes**	mv	median	std	n	MMU2	MMU9	MMU11	MMU14
MMU1	85.0	83.9	48.5	34	0.254	0.608	0.728	0.185
MMU2	95.3	105.6	46.9	32		0.964	0.176	0.044
MMU9	99.5	105.5	44.8	32			0.146	0.163
MMU11	79.4	89.4	44.4	41				0.532
MMU14	71.7	69.9	50.5	34				

								

**fibroblasts**	mv	median	std	n	MMU2	MMU9	MMU11	MMU14

MMU1	77.5	69.8	58.4	26	0.023	0.137	0.061	0.303
MMU2	103.4	118.0	43.0	30		0.586	0.512	0.682
MMU9	102.5	103.1	46.5	30			0.783	0.434
MMU11	112.2	113.2	42.9	31				0.331
MMU14	92.2	97.4	43.7	26				

								

**ES cells**	mv	median	std	n	MMU2	MMU9	MMU11	MMU14

MMU1	101.4	99.2	49.1	25	0.605	0.564	0.182	0.468
MMU2	82.0	90.8	55.0	30		0.388	0.494	0.981
MMU9	98.8	102.0	40.9	30			0.761	0.729
MMU11	97.5	100.6	32.5	22				0.899
MMU14	89.0	90.5	50.6	25				

								

**macrophages**	mv	median	std	n	MMU2	MMU9	MMU11	MMU14

MMU1	46.8	57.6	46.5	20	0.169	0.18	0.003	0.035
MMU2	84.2	87.1	44.2	18		0.999	0.081	0.866
MMU9	81.1	81.0	52.9	17			0.184	0.929
MMU11	109.1	126.3	52.9	30				0.059
MMU14	89.7	106.1	52.4	20				

The model of parental genome separation with a deterministic antiparallel order of chromosomes in the parental chromosome sets [36] requests that angles between given heterologous chromosome territories vary within narrow limits. In a comparison of all "heterologous" angles between two pairs of chromosomes, we would expect a distinct bimodal distribution of these heterologous angles. A fixed smaller angle (Figure 5a, A1-B1 and A2-B2) and a fixed larger angle (A1-B2 and A2-B1). In a cumulative frequency distribution histogram such peaks would cause two sharp increases in the curve at the respective angles (Figure 5b). For different pairs of chromosome territories, fixed but different heterologous angles would be expected, reflecting the different positions of individual chromosome territories in haploid sets with antiparallel order. We measured angles between heterologous chromosomes for the pairs MMU1-MMU14, MMU2-MMU9 and MMU11-MMUX. For a given nucleus, all four heterologous angles (two where MMUX was involved) were calculated if all four territories (three with MMUX) could be segmented. In contrast to the Nagele model [36], all curves for measured heterologous angles showed a very steady increase (Figure 5d,f,h,j), arguing for a very variable side-by-side distribution of chromosome territories. Angle distributions between heterologous chromosomes are very similar for different pairs of chromosomes and we could not detect significant differences in the studied cell types (p > 0.1). We conclude that measured angles between heterologous chromosomes are incompatible both with the hypothesis of parental genome separation and with an antiparallel order of the two haploid sets.

### The spatial organization of chromocenters is dynamic and cell type specific

**Figure 6:**
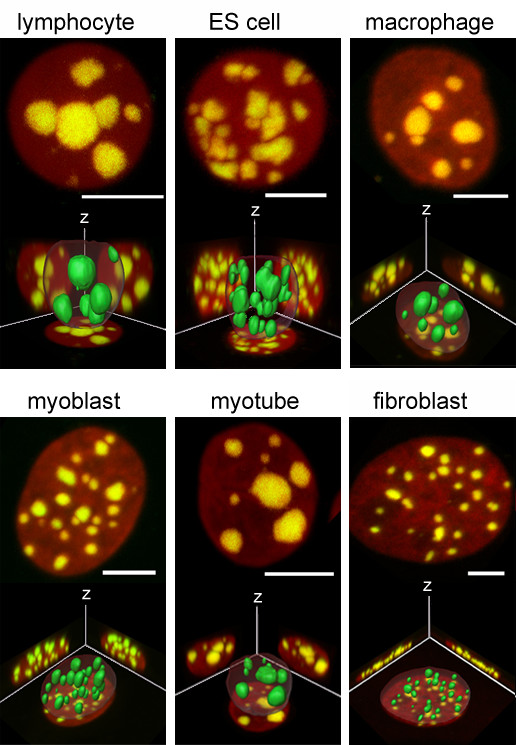
3D-FISH with the mouse major satellite DNA probe. For each cell type, maximum intensity xy-projections (top) and 3D-reconstructions (bottom) are shown. In the projections, DNA counterstain (TO-PRO3) is shown in red and FISH signals are false-colored in green. Overlap of both signals in chromocenters leads to the intense yellow color. Scale bars represent 5 μm in the respective projection. The 3D-reconstructions (not to scale) are shown together with xy, xz and yz maximum intensity projections in the background. Chromocenters in the reconstructions are shown as solid green structures, while the nuclear border is presented as a transparent shell. Note the differences in number and size of chromocenters in the various cell types and the differences in nuclear shape.

**Figure 7:**
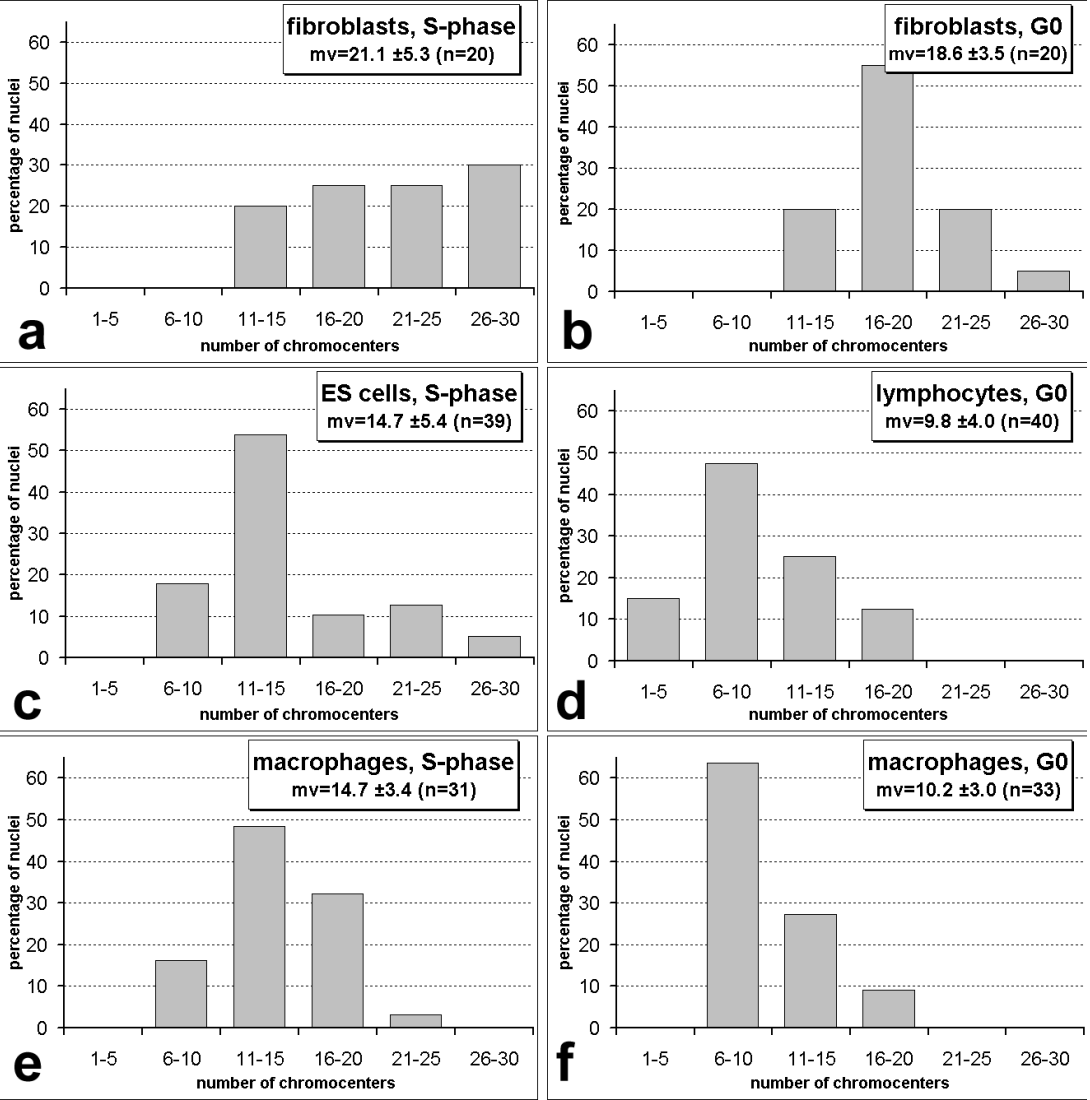
Numbers of chromocenters per nucleus. Cell types are as indicated. mv = mean value, the standard deviation and the number of nuclei (n) are also given.

In mouse interphase nuclei centromere regions cluster to a different degree according to cell type [23-25,29,37], thereby generating so called chromocenters. The mouse major satellite is a pericentromeric satellite DNA that is present in all mouse chromosomes except the Y-chromosome [38]. In nuclei subjected to FISH with the mouse major satellite as a probe (Figure 6) we first counted the number of chromocenters per nucleus. The strongest clustering reflected by the smallest numbers of chromocenters was found in lymphocyte nuclei, the least clustering was observed in fibroblast nuclei (Figure 6, Figure 7; difference highly significant, p < 0.001). Serum-starved G0 fibroblasts did not show a significant difference when compared to S-phase fibroblasts (p > 0.2). During the differentiation of ES cells to postmitotic macrophages, the number of chromocenters decreased significantly (p < 0.01) from an average of 14.7 to 10.2. Post-mitotic macrophages were identified by the presence of CD11b surface antigen and the absence of BrdU incorporation after a 24 hour incubation. We occasionally observed adherent CD11b positive cells that had incorporated BrdU. Some of these cycling macrophage precursors were possibly in their last round of S-phase before entering the postmitotic stage. When we counted the number of chromocenters (average 14.7; Figure 7e) we found a significant difference to postmitotic macrophages (p < 0.001) but not to ES-cells (p > 0.2). This suggests that increased clustering of chromocenters in macrophages occurred during postmitotic terminal differentiation. We previously described a highly significant (p < 0.001) reduction of chromocenter numbers during the differentiation from myoblasts (mean number of 20.4) via post-mitotic myocytes (14.5 chromocenters) to myotubes (11.1 chromocenters) [29].

**Figure 8:**
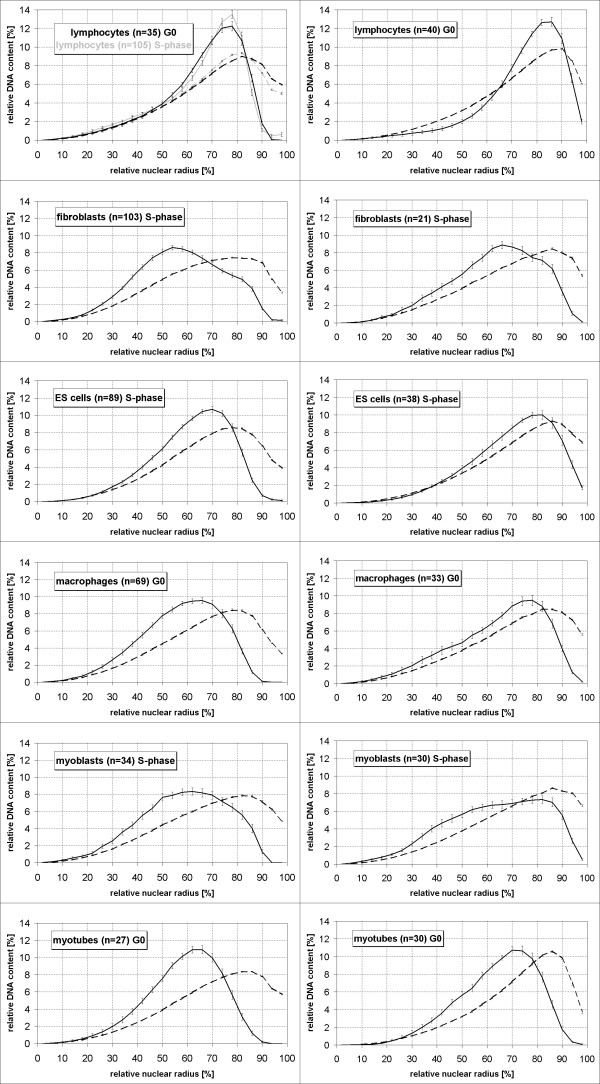
Three-dimensional relative radial distribution of chromocenters (continuous lines) compared to DNA counterstain (broken lines; see legend to Figure 3 and Methods for details). Chromocenters identified by high thresholds of the DNA-counterstain TO-PRO-3 (left) gave results very similar to chromocenters labeled by FISH with a mouse major satellite probe in independent experiments (right).

In contrast to human cell nuclei, chromocenters in mouse cell nuclei are easily identifiable by their extremely bright fluorescence in formaldehyde-fixed, structurally preserved cells counterstained with DAPI or TO-PRO-3 (Figure 2). Our images revealed that these bright areas were identical with the chromocenter FISH signal (Figure 6), except for occasional small parts of counterstained chromatin which escaped detection by FISH. This finding opened the opportunity to investigate the relative radial distribution of chromocenters in the same nuclei that were used for the analysis of the radial chromosome territory distribution. For this purpose we applied a threshold to segment the nucleus and another much higher threshold to segment the intensely stained chromocenters. In all cell types studied, chromocenters had a more internal average position than total nuclear counterstain (Figure 8). The most internal average position was found in fibroblast nuclei, the most peripheral in lymphocyte nuclei. Chromocenter-DNA in quiescent lymphocytes (G0) showed a tendency for a more internal nuclear location compared with S-phase lymphocytes but the difference was not significant (p = 0.68). Differences between both lymphocyte populations and any other cell type were highly significant (p < 0.001). Chromocenter distribution in ES-cells was significantly different from fibroblasts, myoblasts, macrophages (all p = 0.001 or smaller) and myotubes (p = 0.024). A significant difference was also found between myoblasts and myotubes (p = 0.035). Other comparisons revealed no significant differences (p > 0.1). Results obtained with counterstained chromocenters were confirmed by analysis of FISH-labeled chromocenters (Figure 8).

**Figure 9:**
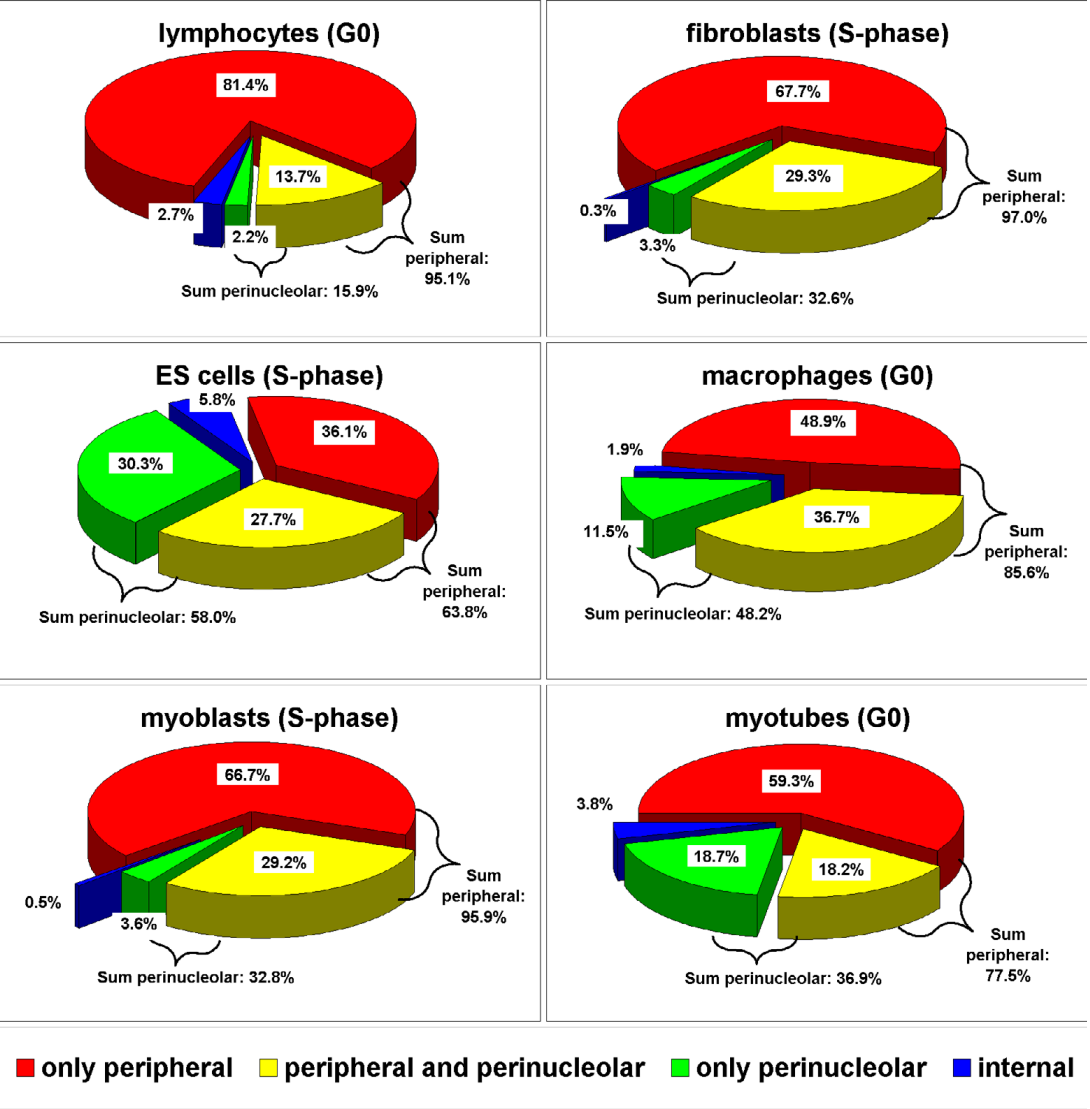
Association of chromocenters with the nuclear periphery and the nucleolus. Pie slices represent the percentages of chromocenters at the respective intranuclear locus in the indicated cell type. See main text for details.

To verify whether chromocenters are in contact with the nuclear border we determined the position of FISH labeled chromocenters (Figure 6) by visual inspection of light optical sections. Each chromocenter was classified to be either in contact with the nuclear border (peripheral), the nucleolus (perinucleolar), both these structures or neither of them (="internal"; Figure 9). In all investigated cell types, the majority of chromocenters were in contact with the nuclear border (64%–97%). A variable fraction was either additionally (14%–37%) or exclusively (3%–30%) touching a nucleolus. The percentage of chromocenters belonging to this perinucleolar fraction varied substantially between cell types (16%–58%). Only a minor fraction of chromocenters was located "internally", i.e. associated neither with the nuclear border nor with a nucleolus (0.3%–6%).

### Nuclear Shapes depend on cell types

Nuclei of the cell types investigated in this study differed substantially in shape (Figure 6). While nuclei from lymphocytes and separately growing ES-cells were approximately spherical, nuclei from fibroblasts and myoblasts resembled flat ellipsoids. Nuclear shape differences may be a factor influencing higher order chromatin arrangements. To relate our above results to nuclear shape we determined the nuclear flatness of all analyzed cell types. For this purpose, we measured in a subset of nuclei the length of the longest nuclear axis in xy-projections which was defined as x-axis, the longest axis that was perpendicular to the x-axis (defined as y-axis) and the z-axis (measured on xz and yz projections of the nucleus). The flatness of the nuclei was then calculated according to (√(x*y))/z (Table 2). A sphere has a value of 1 whereas larger values are obtained for flat structures. As expected, nuclei of ES-cells and lymphocytes revealed the lowest and the very flat fibroblast nuclei revealed the highest values. The difference observed between S-phase and G0 fibroblast nuclei did not result from a difference in nuclear height but from a reduced xy-size of G0 compared to S-phase nuclei. (Table 2). Notably, nuclei may become more spherical or flatter during cell differentiation. While nuclei from in vitro differentiated macrophages were flatter than those from their ES-cell precursors, myotube nuclei were rounder than those from their myoblast precursors.

**Table 2:** Shape parameters of nuclei in different cell types, sorted by increasing flatness. All lengths are in μm and the standard deviation of the mean is given. See text for details

	n=	x-axis	y-axis	z-axis	flatness (√(x*y))/z
ES cells (S-phase)	37	11.5 ± 0.7	10.6 ± 0.7	10.9 ± 1.1	1.01
Lymphocytes (G0)	40	9.2 ± 0.4	8.7 ± 0.4	8.6 ± 0.6	1.04
Myotubes (G0)	31	12.5 ± 2.9	8.2 ± 1.0	7.4 ± 1.1	1.37
Macrophages (G0)	33	13.1 ± 1.4	9.7 ± 1.2	5.8 ± 0.7	1.94
Myoblasts (S-phase)	30	16.2 ± 1.9	11.2 ± 1.6	4.9 ± 0.7	2.75
Fibroblasts (G0)	20	16.0 ± 2.7	11.5 ± 1.6	3.7 ± 0.6	3.67
Fibroblasts (S-phase)	20	19.6 ± 2.5	15.4 ± 1.9	3.8 ± 0.5	4.57

## Discussion

### The radial distribution of chromosome territories

In lymphocyte nuclei of humans, gene rich chromosome territories were shown to locate to internal regions of the nucleus while gene poor ones are more peripheral [11-13,39,40], a distribution also found for the homologs of human chromosomes 18 and 19 in primates [14]. Our data provide the first report for a gene density dependent radial chromosome territory arrangement in lymphocytes of a non-primate animal, suggesting that this ordering principle in the lymphocyte nucleus has been evolutionary conserved since a common ancestor of mice and humans lived some 87 million years ago [41]. The finding that differences are less pronounced in mouse than in humans is consistent with much smaller differences in chromosomal gene density in the mouse karyotype. The evaluation method used here was previously applied in studies on lymphocytes of humans and other primates and results are thus comparable. The most gene rich mouse chromosome MMU11 (peak at 66% of the nuclear radius, Figure 3a) is not as centrally located as the most gene rich human chromosome, HSA19 (peaks at 40–50%) [12,14,40], or the HSA19 homologs in ten primate species (peaks between 40 and 60%) [14] but it comes close to the second most gene rich human chromosome, HSA17 (peak at 58%) [39], which consists of about 3/4 of sequences syntenic to MMU11 [30].

Gene density of individual chromosomes was not the only theme of radial nuclear order, since in addition we observed a correlation with chromosome size (Figure 3). Interestingly, in mouse lymphocyte nuclei the correlation coefficient was higher for a gene density dependent arrangement while in macrophage nuclei it was higher for size dependent arrangement. This indicates a level of cell type specific differences in chromosome territory arrangements whose functional significance can now be explored. Studies finding the same transgene arrays more internal when transcriptionally active than when inactive [42,43] suggest that "gene density sorting" may be correlated to transcriptional activity rather than gene content per se. Current evidence argues against movement of chromosome territories during interphase but repositioning of chromosomes relative to each other was observed during mitosis [3,4].

A comparison of the radial positioning of two chromosomal subregions between human ES and lymphoblastoid cells revealed a slightly significant difference (p < 0.04) for the p-arm of HSA12 but not the p-arm of HSA6 [44]. In our study, between mouse ES cell and lymphocytes nuclei we found many significant differences in the radial distribution of the six tested chromosomes. Differences in fibroblast and macrophage nuclei were much less pronounced. For human fibroblast nuclei, both, a gene density related distribution [11,13,15] and a chromosome size dependent distribution [12,16,17] have been reported. In a recent study [17], we reconciled these seemingly conflicting data by evidence that both, gene density and size related features of chromosome territory positioning can be observed (see Introduction). Territories of small HSA18 and HSA19 were both found close to the 3D nuclear center in spite of the large differences in gene density between them. HSA1 is about 3.5 times larger than HSA18 and HSA19. The largest ratio in the current study was only 1.64 (MMU1 vs. MMU14). A similar factor is reached for example by the human chromosome combinations HSA1 and HSA8 or HSA12 and HSA18. Both combinations were not found to produce significant radial positioning differences in human fibroblasts [17]. Assuming that chromosome size differences play an important role in chromosome territory positioning in both, human and mouse nuclei, the much smaller size differences between mouse chromosomes compared to human chromosomes may explain the lack of significant radial distribution differences in mouse fibroblasts. Linear regression analysis showed a slightly better fit for a gene-density related distribution than for a size related distribution in this cell type (Figure 3e,f).

A chromosome territory distribution related to both, size and gene density was also reported for chicken cell nuclei [7,19]. This fits with the fact that chicken microchromosomes show a much higher gene density than macrochromosomes. In addition, in species ranging from humans and other mammals to chicken, a layer of chromatin at the nuclear periphery and around nucleoli is replicated in mid to late S-phase and consists of gene poor sequences. Gene dense chromatin replicates early in S-phase and is distributed in interior nuclear zones between the perinucleolar and perinuclear compartments [7-9]. Two recent studies found early replicating chromatin also in the interior of Hydra cell nuclei [6] and of micronuclei of a Ciliate [5], while a zone of mid-late replicating chromatin was noted in close association with the nuclear envelope. While it is not known at present whether early and mid-late replicating chromatin in Hydra and Ciliates differ in gene density to the same extend as observed in higher animals, present data support the hypothesis that non-random radial chromatin arrangements have been evolutionary conserved possibly since the formation of the first eukaryotic cells. This hypothesis, if it can be further substantiated, argues for a still unknown adaptive value of this radial order [17,45].

Despite relatively small gene density and size differences between mouse chromosomes we found significant variations in distribution from one cell type to another. The strongest case was provided by the comparison of ES cell nuclei with lymphocyte nuclei where we detected significant differences for MMU9, MMU11 and MMU14. Both mouse cell types have very similar nuclear shapes. We therefore can exclude that these distribution differences are strictly dependent on a single factor, be it nuclear shape, chromosomal size or gene density. More complex mechanisms must therefore be implicated. A possibility that is now open for experimental tests are cell type specific differences of gene expression pattern along a given chromosome. A more similar chromosomal distribution in related cell types than in unrelated ones provides circumstantial evidence for such an assumption [18]. In mouse large and small lung cells the distribution of all tested chromosomes was similar and mouse lymphocytes and myeloblasts showed only one significant difference [18]. In our study we found highly significant differences between two hematopoietic cell types, lymphocytes and macrophages, suggesting that terminal differentiation implies cell type specific changes of chromosome positioning, possibly in response to transcriptional changes. Restrictions for the spatial distribution of chromosome territories may come from the arrangement of specific chromosomal subregions such as pericentromeric heterochromatin which may be involved in the development of cell type specific higher order chromatin arrangements [46].

### Arrangements of chromosome territories follow probabilistic rules

Radial distributions as discussed above were derived as a mean of the positions found in individual nuclei. In individual nuclei, chromosome territories can occupy a position quite different from this mean, reflecting the dynamic nuclear organization of the genome. Notably, chromosome territories were considerably more variable arranged with respect to each other than with respect to their radial nuclear order. Our data are neither compatible with a general association of homologous chromosomes nor with a separation of the genome in two parental haploid sets. A spatial separation of paternal and maternal haploid chromosome sets together with an antiparallel chromosome order in each set resulting in homologous chromosomes typically positioned opposed to each other was reported [36,47-49]. Other studies, however, did not find evidence for these claims in human cell types [17,50]. The present study provides substantial evidence against separation of paternal and maternal chromosome sets in several mouse cell types. Instead, our data support a very variable distribution of chromosome territories with respect to each other, in agreement with a study of radiation induced chromosome translocations in human lymphocytes [51]. Our findings, however, do not exclude preferential neighborhoods of certain chromatin regions in specific cell types. A number of publications have reported individual examples for such a non-random proximity of particular chromosomes [18,52], centromeres [53,54] or genes [55-59] in some cell types but not in others, including homologous pairing of specific chromosomes in some examples [60-62]. This study suggests a more frequent association of MMU1 in macrophage nuclei compared to other cell types.

### Organization of chromocenters

Our results confirm previous studies on other mouse cell types showing characteristic cell type specific patterns of chromocenter distribution [20,24-26,29,37,63,64]. Similar observations have been made in rat [65] and human cells [44,53,54,60,66,67]. The extend of centromere clustering is, however, also species specific. Human fibroblasts, lymphocytes, and ES cells, revealed more than 30 centromere signals in cycling cells for the 46 human chromosomes [44,67] and thus much less clustering than the respective mouse cell types in our study. In addition to the number of chromocenters, the present study also provides data about their radial distribution, their association with the nuclear border or the nucleolus and the shape of the harboring nuclei. As for chromosome territories, we found common themes. Cell types with spherical nuclei revealed a more peripheral relative radial distribution of chromocenters while flat nuclei showed a more internally located one.

With the exception of gene richest MMU11 in some cell types, radial distributions of investigated chromosome territories were more peripheral than the distributions of counterstained nuclear DNA (Figure 3a,d,g,j,m), including chromosomes MMU2 and MMU9, the fifth and sixth gene richest chromosomes in mouse. This raised the question which chromosomes or parts thereof account for the internally located DNA. The 14 chromosome pairs not investigated in this study including six pairs of NOR bearing chromosomes come into question as well as chromosome regions not detected by FISH with chromosome paint probes. In chromosome painting experiments, repetitive sequences that would cross-hybridize to other chromosomes are suppressed. As a consequence, tandem repetitive sequences contained in centromeric and pericentromeric regions stay unlabeled. Indeed, chromocenters were more internal than the average painted chromosome territories from the same cell nuclei and also than total counterstained DNA. Considering the large size of chromocenters, this finding is compatible with the observation that in all cell types 64% – 97% of the chromocenters touched the nuclear border. 3D-reconstructions (Figure 6) illustrate several examples where chromocenters touch the nuclear border but also reach deep into the nuclear interior. This figure also suggests a reason for the tendency of flat cells to have more and smaller chromocenters (Table 2, Figure 7). The average chromocenter in fibroblast nuclei contained pericentromeric regions from two chromosomes. For geometrical reasons, the number of chromosome territories of which centromeres can associate within single chromocenters may be more constrained in flat nuclei compared to spherical nuclei.

Nuclear shape however cannot be the only reason for differences of higher order chromatin arrangements between cell types since nuclei with similar shape but from different cell types such as ES cells and lymphocytes show marked differences. Also, the number of chromocenters is not always larger in flatter nuclei. When ES cells were in vitro differentiated to macrophages their flatness increased while the number of chromocenters decreased. The finding that in this case centromere clustering happens during a postmitotic stage, argues for a differentiation related process. In the differentiation pathways investigated in the present study (myoblasts to myotubes and ES cells to macrophages), we found a decrease in the number of chromocenters. Such a relation was noted in an early study using Giemsa staining on different tissues of mouse [37] and also found in in vitro differentiation experiments [29,65,66]. Generally, non-cycling cells often show fewer chromocenters than their cycling counterparts [33,67]. The extreme case is reached in certain neuronal cells of the mouse retina were all centromeres cluster into a single chromocenter (I. Solovei, personal communication). Our observations suggest, however, that cell differentiation in other cases may also imply a de-clustering of centromeres. Fibroblast and myoblasts nuclei showed larger numbers of chromocenters than ES cell nuclei. More direct evidence is available for postmitotic mouse Purkinje neurons where clustering of centromeric regions is dynamic during postnatal development. After a transient increase in clustering combined with a more central location 3 days after birth, a fraction of centromeric regions split up again together with some centromere movements back to the nuclear periphery [23-25].

## Conclusion

We report common themes of higher order chromatin arrangement as well as cell type specific differences in mouse cell nuclei. A common theme detected here as well as in previous studies of human cell nuclei [11-13] is the preferential radial distribution of chromosome territories, that describes the distance of territories to the nuclear center. In both, human and mouse lymphocyte nuclei, gene rich chromosome territories are distributed to more internal regions than gene poor chromosome territories, indicating evolutionary conservation of this ordering principle at least since the separation of primate and rodent ancestors. In all investigated mouse cell types, we observed a tendency for such a gene density dependent distribution of chromosome territories as well as a preference of large territories to be more peripheral than small ones. Cell type specific differences however were noted with respect to the predominance of gene density or size related correlations. In addition, individual chromosome territories showed cell type specific variations in radial distribution. Cell type specific higher order chromatin arrangements could not be explained by differences in nuclear shape and thus other yet unknown factors must be implicated. In contrast to the radial distribution of chromosome territories in the nucleus, their side-by-side arrangements (neighborhoods) were highly variable. Our data are not compatible with a reported model of separation of haploid parental chromosome sets with an antiparallel order of chromosomes [36]. Depending on cell type, clustering of centromeric regions into larger chromocenters was either increased or decreased compared to precursor cells. In general, we found stronger clustering in further differentiated cells as well as in spherical nuclei when compared to flat nuclei but exceptions occurred. Cell type dependent variations also included differences in radial nuclear distribution of chromocenters. A common theme was contact of a majority of chromocenters with the nuclear border.

## Materials and methods

### Cell culture, fixation procedure and FISH-pretreatments

EB-5 ES-cells (40, XY) were cultivated in DMEM with 15% FCS (tested for ES-cells) with additional supplements as described elsewhere [68] under 5% CO2. The ES-cells grew in gelatinized flasks without feeder cells. For 3D-preparations, glass cover slips were coated with gelatine (pork skin gelatine, Sigma, Deisenhofen, Germany) by incubation with a 1% solution in water for 20 min and air drying. ES-cell suspension was incubated for 1 h to allow attachment. When ES cells grow on a surface for extended periods of time they start to form colonies in which the cells can have nuclei of highly irregular shape. For technical reasons we limited our evaluations to single cells with round nuclei. Differentiation of ES cells to macrophages was started by co-cultivation of ES cells on OP9 stroma cells [69] as described in [70]. At day 8 of differentiation, suspension cells were transferred to cell culture flasks using medium containing macrophage colony-stimulating factor (MCSF) and interleukin 3 (IL-3). Cytokines were obtained by cultivation of L-cells and X63 AG-653 cells, transgenically expressing and secreting M-CSF or IL-3, respectively [71]. On day 12, the culture contained many adherent macrophages. Cells were transferred onto glass coverslips and fixed the following day. Terminally differentiated macrophages were identified by detection of the surface antigen CD11b, by cell shape and by the absence of BrdU incorporation (see below). Mouse embryonic fibroblasts (40, XX and 40, XY, kindly provided by Dr. Alexander Pfeifer, Institut für Pharmakologie, Ludwig-Maximilians-Universität München) were cultured in DMEM with 10% fetal bovine serum under 5% CO2 to 80% confluency on glas coverslips. Mouse lymphocytes from pooled peripheral blood (kindly provided by Dr. Manuela Mohr, Lehrstuhl für molekulare Tierzucht und Biotechnologie, Ludwig-Maximilians-Universität München) were isolated on a Ficoll gradient. Cultivation was in RPMI with 15% fetal bovine serum. Stimulation was with 12 μg/ml concanavalin A for 72 h. After centrifugation, cells were resuspended in 50% FCS/ 50% RPMI. Glass cover slips (18 × 18 mm, 170 μm thick) were coated with poly-L-lysine (MW 300 000, Sigma, Deisenhofen, Germany) by incubation with a 0.1 mg/ml solution for 40 min, washed with water and air-dried. The cell suspension was incubated for 1 h or longer to allow for attachment. Pmi28 primary mouse myoblasts were kindly provided by A. Starzinski-Powitz [72] and cultured and differentiated to myotubes as described [73].

For the identification of cells in S-phase, BrdU at a final concentration of 5 μM was added to the culture medium 30–60 min before fixation except for macrophages and myotubes where incubation time was 24 h. Fixation was performed with 4 % formaldehyde freshly made from paraformaldehyde [74] and buffered in PBS for 10 min. For ES-cells, macrophages, fibroblasts and myotubes, formaldehyde was in 0.75 × PBS, for myoblasts in 1 × PBS. Lymphocytes were incubated in 0.3 × PBS for 40 sec prior to fixation and also fixed in 0.3 × PBS to prevent shrinkage of the nucleus that otherwise occurs in this cell type. Permeabilization steps for all cells included 10 min in 0.5% Triton-X 100, 60 min. incubation in 20% glycerol in PBS followed by five freezing/thawing cycles in liquid nitrogen and a 10 min incubation in 0.1 M HCl. Slides were kept at 4°C in 50% formamid/2 × SSC until hybridization. Air-drying of nuclei was carefully avoided at all steps.

To avoid obstruction due to mixed results from active and inactive X-chromosomes, we investigated only active X-chromosomes from male cells. ES cells, unstimulated lymphocytes and myoblasts were from male sources, thus they as well as in vitro differentiated macrophages and myotubes contained only an active X-chromosome. Fibroblasts and stimulated lymphocytes were from mixed female and male sources. When we labeled X-chromosomes in these cell types, only nuclei with a single one-chromosome-size territory (male cells) were recorded.

### DNA probes and FISH

Mouse chromosome paint probes, produced by DOP-PCR [75] from sorted chromosomes, were kindly provided by N. Carter, Cambridge, UK [76]. Labeling of chromosome paints was done by DOP-PCR using biotin-dUTP or digoxigenin-dUTP. 10 μl of both chromosome paint probes and 80 μl mouse C_0_t1-DNA (1 μg/μl, Invitrogen) were precipitated and solved in 5 μl deionized formamide. The same volume of 20% dextransulphate in 2 × SSC was added. Simultaneous denaturation of probes and target was at 75°C for 3.5 min. Hybridization was performed at 37°C for 2–3 days. To exclude influences from the labeling scheme we switched biotin and digoxigenin so that half of the evaluated nuclei had one labeling scheme and the other half the other one. FISH with the mouse major satellite specific probe was performed as described [29].

### Detection

After hybridization washing steps with 2 × SSC at 37°C and 0.1 × SSC at 60°C were performed. Biotin was detected with avidin-Alexa-488 (Molecular Probes, USA) and goat-anti-avidin-FITC (Vector Laboratories, USA). Digoxigenin was detected with rabbit-anti-Dig (Sigma) and goat-anti-rabbit-Cy3 (Amersham Pharmacia, UK). BrdU detection was in PBS with mouse-anti-BrdU (Roche, Mannheim, Germany) and goat-anti-mouse-Alexa-350 (Molecular Probes, Eugene, Ore.). TO-PRO-3 (1 μM; Molecular Probes) was used as a DNA counterstain.

### Confocal microscopy and image analysis

Stacks of optical sections were collected on Leica TCS 4D (100x, N.A 1.4 Plan Apo Objective) and on Zeiss LSM 410 (63x/1.4 Plan Apo) confocal microscopes. Voxel size was 80 nm or below in xy and 240 nm or below in z. Where necessary, individual image stacks were processed with ImageJ [77] e.g. to clip other nuclei from the images. The program used to determine the relative radial distributions of chromosomes and chromocenters is described in detail elsewhere [12]. Briefly, it segments each nucleus in 25 equally spaced "shells". The outermost shell is fitted to the surface of the segmented nucleus and inner shells are adapted accordingly. On any ray from the nuclear center to the surface, each shell has the same width, resulting in increasing volumes for outer shells. The percentage of a given signal in each shell is then calculated. Due to the limited resolution of light microscopy and a Gaussian filtering, the edge of the nucleus does not appear as a sharp border but blurred, with intensity decreasing to zero over a small region. Nuclear segmentation will include some of it. This is the reason for the decreasing amounts of DNA in the outermost shells in the curves. Angles and distances between chromosome territories were measured with a newly developed program. Thresholds for nuclei and territories were determined interactively. The gravity centers of the resulting objects and the geometrical center of the nucleus were used for calculations. 3D reconstructions shown in Figure 6 were made with AMIRA (TGS Europe, now available from Mercury Computer Systems, Merignac, France). Graphs were made in Microsoft Excel. Final figures were assembled in Adobe Photoshop (Adobe Systems, San Jose, CA, USA).

### Statistical analysis

To determine whether differences between relative radial distributions were significant, we used the median of the distribution in each nucleus. These and other values like angles were compared using the two-sided Kolmogorov-Smirnov test in the Software Package SPSS 12 (SPSS Inc., Chicago, Ill.). Linear regression analysis was also performed in SPSS.

## Authors' contributions

This study was conceived and supervised by SD and TC. RM and AB performed the experiments and quantitative evaluation. TS helped setting up the ES cell differentiation. JvH wrote the quantitative evaluation programs which were embedded in a shell script environment by SD. Statistical evaluation was performed by AB, RM and SD. The manuscript was written by SD with contributions from TC and help from all coauthors.

## Supplementary Material

Additional File 1 All median values of the relative radial distribution of chromosome territories in individual nuclei (see legend to Figure 3). Values in this text file mediantable.txt are separated by tabs. Click here for file
